# Exogenous feeding of immediate precursors reveals synergistic effect on picroside-I biosynthesis in shoot cultures of *Picrorhiza kurroa* Royle ex Benth

**DOI:** 10.1038/srep29750

**Published:** 2016-07-15

**Authors:** Varun Kumar, Neha Sharma, Hemant Sood, Rajinder Singh Chauhan

**Affiliations:** 1 Department of Biotechnology and Bioinformatics, Jaypee University of Information Technology, Waknaghat-173234, Solan (HP), India

## Abstract

In the current study, we asked how the supply of immediate biosynthetic precursors i.e. cinnamic acid (CA) and catalpol (CAT) influences the synthesis of picroside-I (P-I) in shoot cultures of *P. kurroa*. Our results revealed that only CA and CA+CAT stimulated P-I production with 1.6-fold and 4.2-fold, respectively at 2.5 mg/100 mL concentration treatment. Interestingly, feeding CA+CAT not only directed flux towards p-Coumaric acid (p-CA) production but also appeared to trigger the metabolic flux through both shikimate/phenylpropanoid and iridoid pathways by utilizing more of CA and CAT for P-I biosynthesis. However, a deficiency in the supply of either the iridoid or the phenylpropanoid precursor limits flux through the respective pathways as reflected by feedback inhibition effect on PAL and decreased transcripts expressions of rate limiting enzymes (DAHPS, CM, PAL, GS and G10H). It also appears that addition of CA alone directed flux towards both p-CA and P-I production. Based on precursor feeding and metabolic fluxes, a current hypothesis is that precursors from both the iridoid and shikimate/phenylpropanoid pathways are a flux limitation for P-I production in shoot cultures of *P. kurroa* plants. This work thus sets a stage for future endeavour to elevate production of P-I in cultured plant cells.

*Picrorhiza kurroa* Royle ex Benth, commonly known as Kutki, belongs to family Plantaginaceae (formerly known as Scrophulariaceae) and is distributed at an elevation of 3000–5000 m above mean sea level in the North-Western Himalayas^1^. It is a therapeutically important herb that has a long tradition of being used as herbal drug in the treatment of liver disorders. Its pharmaceutical potential is attributed to the presence of active constituents mainly picroside I (P-I) and picroside II (P-II)^2^. Over the years, the rising demand of picrosides facilitated the overexploitation of this plant species which has reduced its population in natural habitat and put it in the category of endangered plant species^3^. As a result, legal restrictions are imposed on the collection of *P. kurroa* plants from their natural habitats leading to illegal procurement and adulteration due to shortage of raw material for herbal drug industries. Therefore, production of quality plant material with increased picrosides content is the only way to provide sustainable solution to the problem. Nevertheless, plant tissue culture has been standardised for picrosides production but the P-I content is very low in tissue cultured plants compared to plants grown in natural habitats^4^. To address this problem, metabolic engineering has the potential to effectively maximize picrosides content in cellular systems by redirecting the carbon flux towards picrosides biosynthesis. However, this remains a formidable task considering the fact that biosynthetic architecture of picrosides is complex, non-linear and fluxes are poorly understood.

The biosynthesis of P-I proceeds via non-mevalonate (MEP), mevalonate (MVA), shikimate/phenylpropanoid and iridoid pathways^5^. During the past years, numerous efforts have been carried out to shortlist candidate genes involved in P-I production through gene expression analyses performed under differential conditions of picrosides accumulation in *P. kurroa*^6^,^7^,^8^,^9^,^10^,^11^,^12^. Despite interesting cues obtained from these reports, no information is yet available on what mechanism controls the flux of P-I biosynthesis in *P. kurroa*. Furthermore, it is still unclear which out of the different modules is limiting for the P-I supply? It is, therefore, crucial to understand the mechanism behind P-I biosynthesis in *P. kurroa* prior to designing a suitable rational metabolic engineering approach.

We address this issue here through feeding of different precursors in the context of P-I biosynthesis in *P. kurroa*. It has been reported that exogenous feeding of precursors situated at the key biosynthetic steps can stimulate the production of target secondary metabolites in *in vitro* conditions, provided the endogenous levels of selected precursors are a limiting factor for the target metabolite biosynthesis^13^. Several reports have shown the influence of precursor availability on the accumulation of target metabolites and metabolic flux limitations^14^,^15^. Exogenous tryptamine and loganin has been reported to increase the levels of secologanin in transgenic cell lines of *Catharanthus roseus* and utilization of supplied precursors determined the metabolic flux through the pathway components^14^. Moreover, *C. roseus* hairy root cultures were also subjected to exogenous precursors of terpenoid and tryptophan branches to determine the metabolic flux limitations of two branches to indole alkaloids^15^.

In this paper, we report a series of experiments designed to determine the effect of cinnamic acid (CA) and catalpol (CAT) alone and in combinations on the flux limitations leading to P-I biosynthesis in *in vitro* grown shoots of *P. kurroa*.

## Results

### Determination of optimum concentration for different precursor treatments

**Figure 1:**
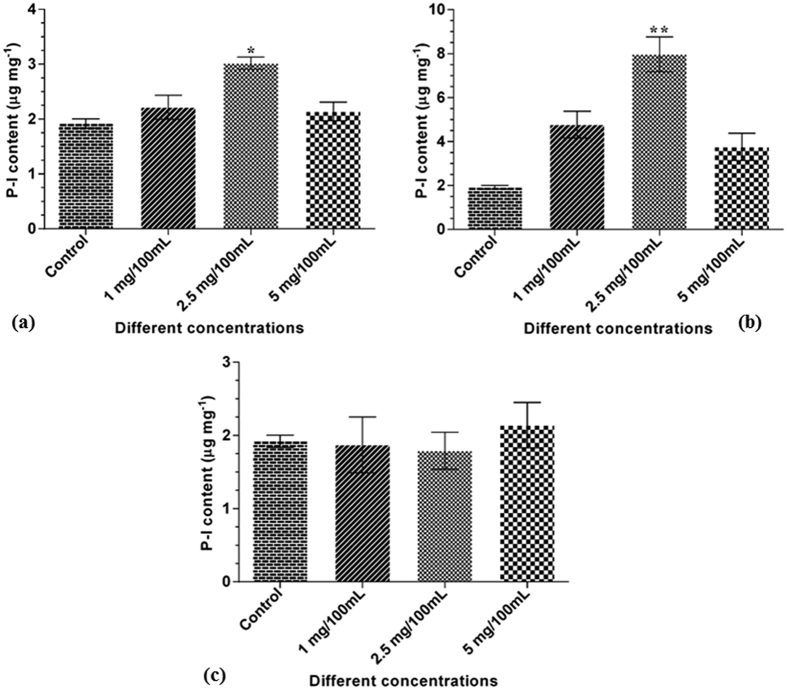
Optimum concentration determination for different precursor treatments; (**a**) CA, (**b**) CA+CAT and, (**c**) CAT. The optimum concentrations were determined by observing their effects on increase in P-I content. The data show means ± SD (n = 3). Significance was evaluated within each feeded samples between different concentrations of treatments and untreated control. (*p < 0.05, **p < 0.01).

Different concentrations (1 mg/100 mL, 2.5 mg/100 mL and 5 mg/100 mL) of CA, CAT and CA+CAT were tested for their effect on P-I content in *in vitro* grown shoots of *P. kurroa* plants. Among the tested concentrations of CA and CA+CAT, significant increment in P-I content was observed with 1.6-fold (*p* < 0.05) and 4.2-fold (*p* < 0.01), respectively at 2.5 mg/100 mL concentration compared to untreated control shoots (Fig. 1a,b). In contrast, the tested concentrations of CAT alone showed no significant increase in P-I content when compared to untreated control shoots (Fig. 1c). It was evident from the results that 2.5 mg/100 mL concentration of CA and CA+CAT effectively maximize P-I content in shoots of *in vitro* grown *P. kurroa* plants and thus, referred to as optimum concentration for further precursor’s treatments. The HPLC chromatograms of P-I standard and the samples are provided in Supplementary Fig. 1.

### Influence of different precursor treatments on shoot biomass, p-Coumaric acid (p-CA) and CA content

**Figure 2:**
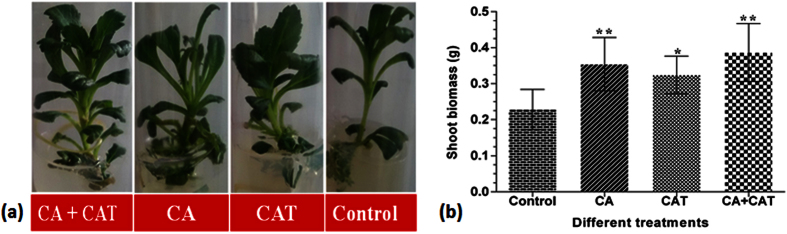
Comparative analysis of different precursor treatments for their effect on shoot biomass; (**a**) *P. kurroa* shoots grown at optimum concentration of different precursors, (**b**) Shoot biomass (g). The data show means ± SD (n = 3). Significance was evaluated between different treatments and untreated control. (*p < 0.05, **p < 0.01).

**Figure 3:**
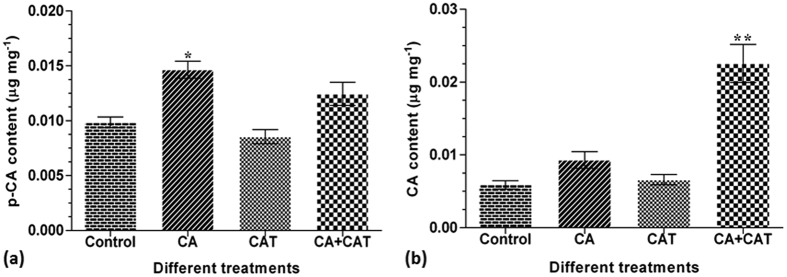
Influence of different precursor treatments on the production of tested metabolites; (**a**) p-CA, (**b**) CA. The data show means ± SD (n = 3). Significance was evaluated between different treatments and untreated control. (*p < 0.05, **p < 0.01).

The *P. kurroa* shoots treated with CA, CAT and CA+CAT at optimum concentration in addition to untreated control shoots were screened for increase in shoot biomass and subjected to p-CA and CA content analysis. The data revealed that all treatments viz. CA, CAT and CA+CAT showed significant increase in shoot biomass with 1.5-(p < 0.01), 1.4-(p < 0.05) and 1.7-fold (p < 0.01), respectively compared to untreated control shoots (Fig. 2a,b). Further, p-CA content showed significant increase with 1.5-fold (*p* < 0.05) in shoots treated with CA while non-significant increase was observed in CAT and CA+CAT treated shoots compared to untreated control (Fig. 3a). In contrast, CA content showed 3.8-fold (*p* < 0.0001) increase in shoots treated with CA+CAT while no increase was observed in CA and CAT treated shoots compared to shoots without treatment (Fig. 3b). The HPLC chromatograms of p-CA and CA standards and the samples are provided in Supplementary Figs 2 and 3.

### Expression status of selected genes in different precursor treatments

**Figure 4:**
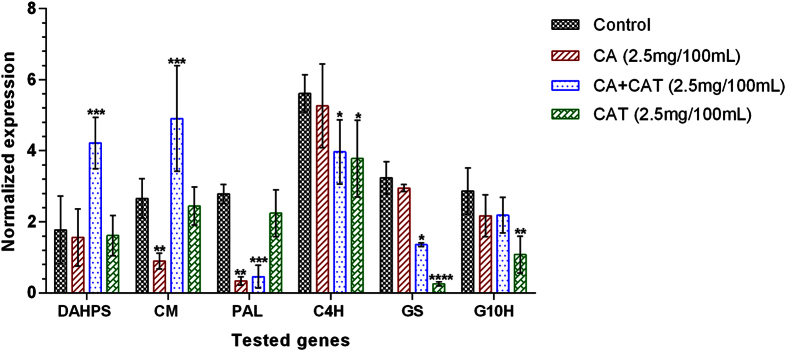
Expression profiles of selected shikimate/phenylpropanoid and iridoid pathway genes in CA, CAT and CA + CAT feeded shoot cultures of *P. kurroa*. Expression levels were normalized to 26S and GAPDH reference genes expressions. Data is reported as average of four replicates ± SD of the mean. Significance was evaluated for each gene between different treatments and untreated control (*p < 0.05, **p < 0.01, ***p < 0.001, ****p < 0.0001).

The transcript levels of shikimate/phenylpropanoid and iridoid pathway genes were checked in CA, CAT and CA+CAT treated shoots of *P. kurroa* due to their involvement in the biosynthesis of respective metabolites. Among the shoots treated with CA+CAT, 3-deoxy-D-arabinoheptulosonate 7-phosphate synthase (DAHPS) and chorismate mutase (CM) were up-regulated by 2.4-fold (*p* < 0.001) and 1.8-fold (*p* < 0.001), respectively whereas phenylalanine ammonia lyase (PAL), cinnamic acid-4-hydroxylase (C4H) and geraniol synthase (GS) were down-regulated by 6.2-fold (*p* < 0.001), 1.4-fold (*p* < 0.05) and 2.4-fold (*p* < 0.05), respectively, compared to untreated control shoots. In contrast, the transcript encoding geraniol-10-hydroxylase (G10H) showed no alteration compared to untreated control shoots (Fig. 4). However, shoots treated with CA showed down-regulation of CM and PAL transcripts with 3-fold (*p* < 0.01) and 8.2-fold (*p* < 0.01), respectively whereas transcripts encoding DAHPS, C4H, GS and G10H enzymes showed non-significant modulation compared to untreated control shoots (Fig. 4).

Further, the shoots treated with CAT revealed down-regulation of C4H, GS and G10H transcripts with 1.5-fold (*p* < 0.05), 12.9-fold (*p* < 0.0001) and 2.7-fold (*p* < 0.01), respectively whereas transcripts encoding DAHPS, CM and PAL enzymes showed non-significant modulation compared to untreated control shoots (Fig. 4).

### Effect of different precursor treatments on PAL activity

**Figure 5:**
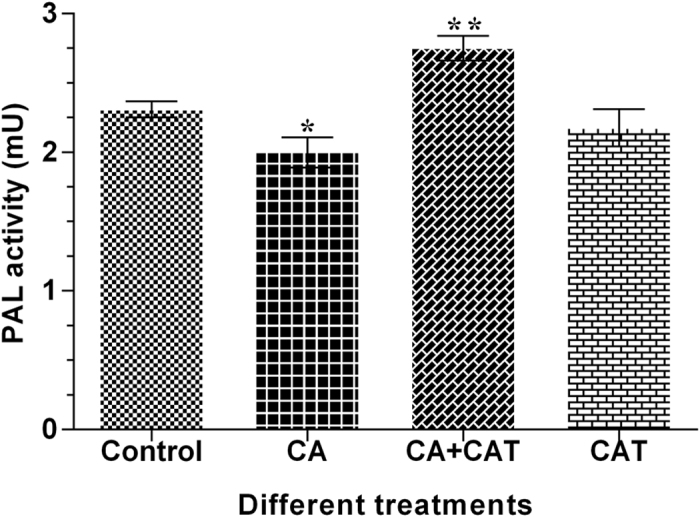
Activity profiles of PAL in CA, CAT and CA+CAT feeded shoot cultures of *P. kurroa*. Data is reported as average of three replicates ± SD of the mean. Significance was evaluated between different treatments and untreated control (*p < 0.05, **p < 0.01).

The analysis of CA, CAT and CA+CAT fed shoots of *P. kurroa* revealed significant variations in the activity of PAL enzyme compared to untreated control shoots. The PAL activity was down-regulated by 1.2-fold (*p* < 0.05) in shoots fed with CA while up-regulated by 1.2-fold (*p* < 0.01) in CA+CAT fed shoots compared to untreated control shoots. In contrast, the activity of PAL enzyme showed non-significant alteration in CAT treated shoots compared to untreated control shoots (Fig. 5).

### Correlation analysis

**Figure 6:**
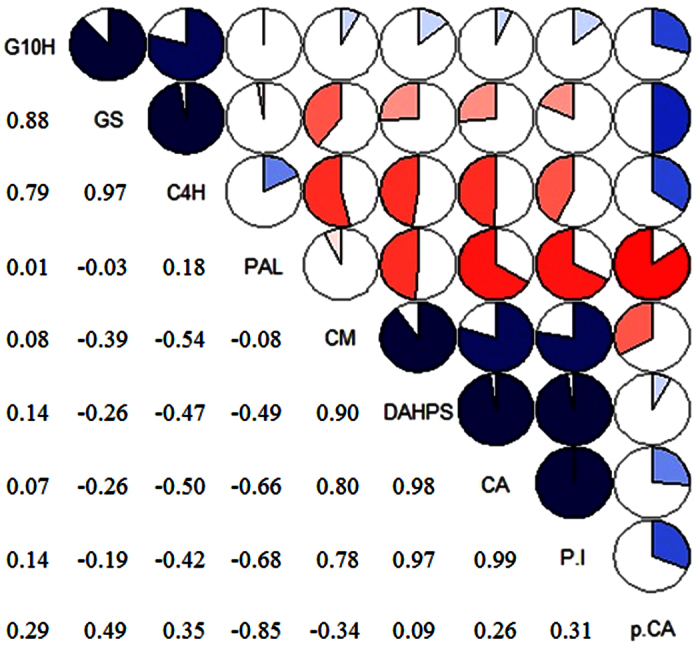
Correlogram showing correlations of tested genes and metabolites data. Pearson’s correlation coefficients in genes and metabolites from the control, CA, CAT and CA+CAT treatment’s group. The data having similar patterns are grouped together and presented in the form of pie graphs filled in proportion to the Pearson’s coefficient values. Blue colored pie graphs filled clockwise indicate positive correlations while red colored pie graphs filled anti-clockwise indicate negative correlations.

To observe correlations among selected genes and metabolites levels between different precursor treatments, correlation analysis was performed and results are presented in the form of a correlogram as shown in Fig. 6. The correlations among selected shikimate/phenylpropanoid and iridoid pathway genes expression and contents of P-I, p-CA and CA metabolites were observed between control, CA, CAT and CA+CAT treated shoot cultures of *P. kurroa*. The P-I showed positive correlation with CA (0.99), DAHPS (0.97) and CM (0.78) while low correlation was observed with p-CA (0.31) and G10H (0.14). In contrast, negative correlations of P-I were observed with PAL, GS and C4H genes between control, CA, CAT and CA+CAT treated shoots (Fig. 6). We have thus been able to assess from the correlations that DAHPS, CM, CA and G10H might play a crucial role in the biosynthesis of P-I whereas PAL and GS could be the potential regulatory sites of P-I biosynthesis in shoot cultures of *P. kurroa*.

## Discussion

**Figure 7:**
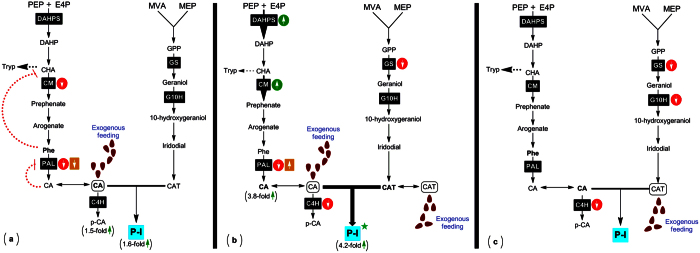
Cascades depicting influence of different treatments on the fluxes through shikimate/phenylpropanoid and iridoid pathways; (**a**) CA, (**b**) CA+CAT and (**c**) CAT. The symbols were added and structures were coded with different colors to highlight the effects occurring on the biosynthesis of P-I under different treatments. Gene expressions were highlighted by using the green circle with arrow pointing up shows up-regulation while red circle with arrow pointing down shows down-regulation. Orange colored box with arrow pointing up and down indicate up-regulation and down-regulation of PAL enzyme, respectively. Red colored loop indicate feedback inhibition effect. Bold arrows indicate the up-regulation of respective step. Green colored star indicate high significant increase. CHA, chorismate; DAHP, 3-Deoxy-D-arabinoheptulosonate 7-phosphate; GPP, geranyl pyrophosphate; PEP, phosphoenolpyruvate; E4P, erythrose-4-phosphate.

**Figure 8:**
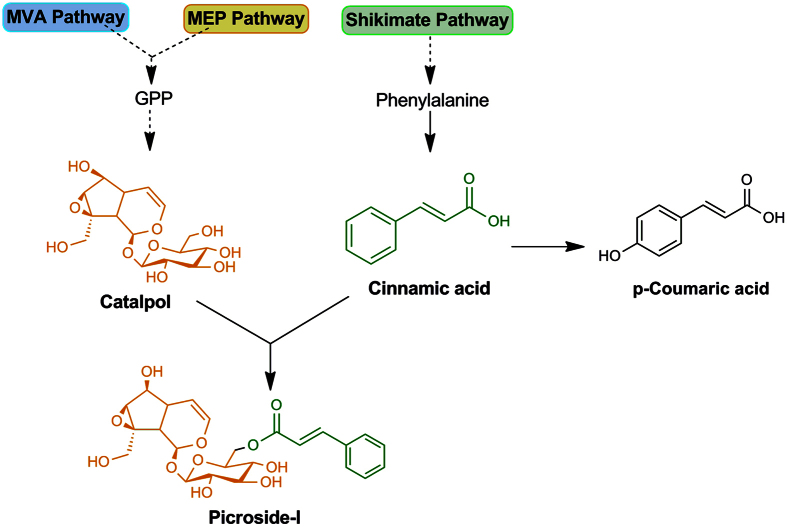
Schematic representation of P-I biosynthesis. The structure of P-I linked to cinnamic acid (green color) and catalpol (orange color) moieties.

To address metabolic basis of P-I biosynthesis in *P. kurroa*, we applied metabolic flux analysis with feeding of late precursors i.e. CA and CAT alone and in combination not only to establish the route of P-I production *in vivo* but also to determine the limiting moiety in the end metabolite. Further, CAT supplied to *in vitro* cultured plants is an iridoid glycoside and hence it is probable that it is not taken up in its present form. Glucosidases may be excreted from cuttings of plants in *in vitro* cultures, which can hydrolyse the catalpol and the resulting aglycon may be taken up by the cultured plants. The results observed in this study showed that exogenous CAT increased P-I content when CA is not limiting indicating that addition of CAT caused rapid utilization of CA due to synergistic effect. Thus, induction of P-I production took place very rapidly after feeding with CA and CAT in combination whereas feeding with CA alone led to a small increase in P-I production (Fig. 7). In contrast to this, CAT feeding showed no increase in P-I content compared to the untreated control. This indicates that abundant supply of both CA and CAT, must exist for high rates of P-I biosynthesis to occur (Fig. 8). Moreover, this also implies that supply of CA is limiting for the biosynthesis of P-I in *P. kurroa*. Whitmer *et al*.^14^ also showed that supply of both tryptamine and secologanin was required for enhanced production of strictosidine in *C. roseus*.

Despite above observations that both shikimate/phenylpropanoid and iridoid pathways act in conjunction for P-I production but are rather limited for designing a suitable rational engineering approach to increase P-I production in *P. kurroa*, as only content of P-I was observed in response to feeding. The CA serves as the branching point for formation of p-CA and P-I in *P. kurroa*^5^. Thus, it is crucial to determine the metabolic fluxes in different treated samples. For this, further experiments were performed. Upon analyses of CA+CAT fed shoots, interestingly, p-CA content showed statistically non-significant increase but CA content showed prominent elevation compared to untreated control. One possibility for this decreased flux towards p-CA compared to P-I is that the exogenous supply of both CA and CAT in combination might increase the activity of probable acyltransferase catalysing the esterification step between CA and CAT to produce P-I. It should be noted that we have not detected the activity of this enzyme, since this probable acyltransferase is still to be identified^8^. Further, the decreased transcript level of C4H in CA+CAT fed shoots might also support the non-significant increase in p-CA production over untreated control. Furthermore, the rapid utilization of exogenous CA for P-I production in CA+CAT fed shoots appears to induce the cells to synthesize more CA as indicated by high CA content. It might direct the flux of shikimate/phenylpropanoid pathway through to the prephenate synthesis step leading to increased Phe availability^16^. This statement was corroborated by enhanced expression of genes encoding DAHPS and CM; enzymes considered to be rate limiting along with PAL for the shikimate/phenylpropanoid pathway^16^,^17^,^18^. CA has been proposed to feedback inhibit PAL activity^18^. Our results showed significant increase in PAL activity which indicates that the rapid utilization of CA might have a de-inhibitory effect on the activity of PAL enzyme (Fig. 7). However, CA+CAT feeding decreased the transcript level of gene encoding PAL. This implies that exogenously supplied CA+CAT exerted both coarse and fine controls to the reaction catalyzed by PAL enzyme. As a whole there are convincing evidences that shikimate/phenylpropanoid pathway stimulate flux during combined augmentation of CA and CAT levels in *P. kurroa*.

Moreover, the analyses of CA fed shoots revealed significant increase in p-CA content but CA content showed non-significant elevation compared to untreated control. It might indicate that the exogenous feeding of CA alone directed the flux both towards p-CA and P-I compared to CA+CAT fed shoots possibly due to the limited CAT supply. Further, the transcript level of C4H showed no statistically significant modulation compared to untreated control. This might indicate that exogenous CA exerted fine control to the reaction catalyzed by C4H enzyme. Apparently, less utilization of CA pool compared to CA+CAT fed shoots appears to inhibit synthesis of endogenous CA as indicated by non-significant increase in CA content compared to untreated control shoots. This statement was in agreement with the results observed which showed significant reduction in PAL activity and gene expression indicating the feedback inhibition of PAL enzyme by both coarse and fine controls. It is likely that accumulation of Phe directs the flux of shikimate/phenylpropanoid pathway to the tryptophan synthesis leading to decreased Phe availability^16^. This feedback regulation in the shikimate/phenylpropanoid pathway occurs at the branching point, chorismate which is the common substrate for CM and anthranilate synthase (AS), enzymes^16^. This statement was corroborated by decreased expression of gene encoding CM enzyme whereas transcript level of DAHPS showed non-significant modulation in *P. kurroa* shoots fed only with CA (Fig. 7).

The CAT fed shoots on the other hand, were found to be associated with non-significant increase in p-CA, CA and P-I content. It might indicate that exogenous feeding of CAT alone is not able to increase the flux both towards p-CA and P-I possibly due to the limited CA supply. Further, the decreased transcript level of C4H in CAT fed shoots might also support the non-significant increase in p-CA production over untreated control. It is likely that CAT fed shoots were accumulating large amount of CAT which might simultaneously limits the flux through the iridoid pathway. To further test the flux limitation, we have detected the transcripts levels of iridoid (GS and G10H) and shikimate/phenylpropanoid pathway (DAHPS, CM, PAL, C4H) enzymes in CAT fed shoots. The above genes of iridoid pathway were selected for the study as it has been reported that these are potential sites for the regulation of iridoid and seco-iridoid production^8^,^12^,^19^. The results revealed decreased transcript levels of GS and G10H which also support the flux limitation through the iridoid pathway in CAT fed shoots (Fig. 7). Further, compared to CAT fed shoots, transcript level of G10H did not show any alterations in CA+CAT fed shoots which might indicate reversed inhibitory effect on the iridoid pathway and increased P-I production in CA+CAT fed shoots. These differences in gene expressions might also ascertain their possible association with the regulation of iridoid pathway.

As a whole in the face of such a complex biosynthetic architecture, one may ask what strategy we should focus on for biosynthetic engineering of P-I in *P. kurroa*. Indeed, we argue that multi-step engineering for augmenting both CA and CAT by exploitation of DAHPS, CM, PAL, GS and G10H catalyzed steps is more prosaic. Several reasons may account for the use of multi-step engineering approach. First, CA is found to be the limiting moiety for P-I production. Second, supplementing both CA and CAT is found to be activating both shikimate/phenylpropanoid and iridoid pathways for enhanced production of P-I whereas their individual application caused feedback inhibition and limits the production of P-I. Third, the combined application of CA and CAT directs maximum flux towards P-I production while CA alone shifts flux towards both p-CA and P-I production. This study thus provides a platform for the improved biotechnological production of P-I in *P. kurroa*.

## Methods

### Plant material

The whole plants of *P. kurroa* procured from nursery of the Himalayan Forest Research Institute, Jagatsukh, Manali, H.P., India (2193 m altitude, 32°12′ 0N and 77°12′ 0E) were maintained in the greenhouse of Jaypee University of Information Technology, Waknaghat, H.P., India (1700 m altitude, 31°0′58.55″ N and 77°4′12.63″ E). The shoot apices of plants were surface sterilized with 0.1% mercuric chloride and 0.5% bavistin (acts as a fungicide) followed by 4–5 washings with sterile water and cultured in an optimized Murashige and Skoog (MS) medium^20^ supplemented with 3 mg/L indole-3-butyric acid and 1 mg/L kinetin. After four weeks of cultivation at 25 ± 2 °C in a plant tissue culture chamber^4^, the cultures were used as the experimental material for treatment with different precursors.

### Addition of different precursors

Solutions of cinnamic acid (CA) (Sigma-Aldrich, USA) and catalpol (CAT) (Sigma-Aldrich, USA) alone and in combination for feeding were prepared as neutral aqueous stocks at three different concentrations *viz.* 1 mg/100 mL, 2.5 mg/100 mL and 5 mg/100 mL. These solutions were filter sterilized directly into culture flasks containing optimized MS medium supplemented with 3 mg/L indole-3-butyric acid and 0031 mg/L kinetin. Shoot cultures of *P. kurroa* grown at 25 ± 2 °C were aseptically transferred to these culture flasks and kept at 15 ± 2 °C in a plant tissue culture chamber. Shoots of *P. kurroa* grown at 15 ± 2 °C without any treatment were used as controls. It has been reported that P-I production is hindered at 25 °C while 15 °C temperature condition increases P-I production in tissue cultured *P. kurroa* shoots^4^. That is why, we took *P. kurroa* shoots grown at 25 °C as an explant and carried out precursor feeding experiments at 15 °C temperature condition. After 30 days of plant growth, the cultures were harvested and shoot samples were immediately stored at −80 °C for analysis of P-I content. The experiment was performed in triplicates.

### Extraction and determination of P-I content

Shoot samples of *P. kurroa* plants collected after treatment with CA, CAT and CA+CAT at three different concentrations along with the untreated shoots, were extracted for P-I in triplicates as per the method described in Kumar *et al*.^7^. The P-I content in the extracts was determined by using HPLC as per the method reported in Pandit *et al*.^21^. The experiment was performed in triplicates.

### Culturing of *in vitro* shoots supplemented with optimum concentration of precursors

The optimum concentration of different precursors was determined after estimation of P-I content in *P. kurroa* shoots treated with different precursors. The concentration of precursors which effectively maximise the P-I content was referred to as optimum concentration. *In vitro* grown *P. kurroa* shoots supplemented with optimum concentration of precursors were then cultured under the same conditions as mentioned above. Shoots of *P. kurroa* plants without any treatment were used as controls. The cultures were harvested after 30 days and data were recorded for total shoot biomass. The shoot samples were immediately stored at −80 °C for further analyses. The experiment was performed three times in triplicates.

### Extraction and quantification of p-CA and CA

For extraction of p-CA and CA, *P. kurroa* shoots without treatment and treated with optimum concentration of CA, CAT and CA+CAT were homogenised in a prechilled mortar and pestle using liquid nitrogen. Each powdered sample (200 mg) was suspended in 2 mL of 100% methanol and 1% BHT; vortexed and sonicated for 30 min at room temperature. The samples were then centrifuged at 10,000 rpm for 10 min. The supernatants thus obtained were filtered through 0.22 μm filter (Millipore), diluted 2.5-fold with 100% methanol and used for quantification of p-CA and CA. The quantification was carried by RP- HPLC (Waters 515) through C18 (5 μm) 4.6 × 250 mm Waters Symmetry Column using PDA detector (Waters 2996). The mobile phase was generated by a gradient elution programme using two solvent systems i.e. Solvent A (1% aqueous acetic acid solution) and Solvent B (methanol). Gradient elution programme was followed as described in Nour *et al*.^22^. Detection of p-CA and CA was carried out at an absorbance of 310 ± 4 nm and 290 ± 4 nm wavelength, respectively. The cycle time of analysis was 48 min at 20 °C. The compound was identified on the basis of retention time and comparison of UV spectra with the p-CA and CA standards (Sigma-Aldrich, USA). The experiment was performed in triplicates.

### Isolation of genomic DNA and total RNA

The shoots of *P. kurroa* plants without treatment and treated with optimum concentration of CA, CAT and CA+CAT were used for isolation of genomic DNA as per the method reported by Murray and Thompson^23^. Total RNA was isolated by using TRIzol reagent (Ambion) as per the manufacturer’s instructions. The quantification of isolated RNA was carried out at 260 nm and 280 nm wavelengths with ND-2000 UV spectrophotometer (Nanodrop Technologies, Wilmington, DE, USA) and quality was checked on 1% (w/v) agarose gel stained with ethidium bromide.

### Complementary DNA (cDNA) synthesis and gene expression analysis

Synthesis of cDNA was performed from 5 μg of total RNA using Verso cDNA synthesis kit (Thermo scientific, US) as per the manufacturer’s instructions. The resulting cDNA was quantified to obtain equal concentration (100 ng) by using ND-2000 UV spectrophotometer. A total of six genes catalysing rate limiting steps in shikimate/phenylpropanoid and iridoid pathways were selected and subjected to quantitative real-time (qRT) PCR analysis. The primers of iridoid pathway genes (GS and G10H) and two genes of shikimate/phenylpropanoid pathway (DAHPS and PAL) were procured from Kumar *et al*.^8^ while rest of the selected genes (CM and C4H) were designed from transcriptomic sequences of *P. kurroa* by using Primer3 software. The details of the primers with annealing temperatures are provided in Table 1. The expression analysis of selected genes was performed in quadruplicates as per the protocol described in Kumar *et al*.^8^ with annealing temperatures varied from 49–59 °C. The housekeeping genes, 26S and GAPDH were used as the standard genes in this study.

### PAL activity assay

PAL was extracted from 0.5 g of *P. kurroa* shoots, both untreated and treated with optimum concentrations of CA, CAT and CA+CAT, by homogenization in a prechilled mortar and pestle using liquid nitrogen with 5 mL of extraction buffer (100 mM sodium borate buffer, pH 8.8, containing 5 mM β-mercaptoethanol). The homogenate was then centrifuged at 10,000 × g for 20 min. The clarified extract thus obtained was referred to as crude extract and used for enzyme assay. The extraction was performed in triplicates.

The standard assay mixture for PAL contained 100 mM sodium borate buffer (pH 8.8), 15 mM phenylalanine (Phe) and 50 μL crude extract in a total volume of 3.0 mL. The mixture without Phe was preincubated at 30 °C for 20 min to allow for an initial non-enzymatic decrease in absorbance and then the reaction was initiated by the addition of Phe. The increase in absorbance was recorded over a period of 60 min at 290 nm wavelength using a UV-VIS spectrophotometer (SPECTRASCAN UV 2700, Thermo Scientific). The PAL activity was determined by standard curve prepared by taking different concentrations of CA. The activity of PAL was expressed as μmol of CA formed/min/mL under the specified conditions and data has been reported as milliunits (mU) of enzyme. The assay was performed in triplicates.

### Correlation analysis

The correlation analysis was performed by calculating Pearson’s correlation coefficients on the gene expression and metabolite content data between different treatments and untreated control. The correlations were displayed as “correlogram” generated by using “Corrgram” function in R package^24^. By this method, correlations were visualised as color-coded pie graphs filled in proportion to the Pearson’s coefficient values.

### Statistical analysis

The statistical analysis was performed from mean ± SD of data in triplicates by using one and two-way ANOVA followed by a Bonferroni test using GraphPad prism software version 6.0.

## Additional Information

**How to cite this article**: Kumar, V. *et al*. Exogenous feeding of immediate precursors reveals synergistic effect on picroside-I biosynthesis in shoot cultures of *Picrorhiza kurroa* Royle ex Benth. *Sci. Rep.* **6**, 29750; doi: 10.1038/srep29750 (2016).

## Supplementary Material

Supplementary Information

## Figures and Tables

**Table 1:** Primer sequences for selected genes used in quantitative RT-PCR analysis.

Genes	Primer sequence	Fragment size (bp)	Annealing temperatures (°C)
**26S**	FP 5′-CACAATGATAGGAAGAGCCGAC-3′RP 5′-CAAGGGAACGGGCTTGGCAGAATC-3′	500	58
**GAPDH**	FP 5′-TTGCCATCAATGACCCCTTCA-3′RP 5′-CGCCCCACTTGATTTTGGA-3′	215	56
**DAHPS**	FP 5′-ACACCATTAAAGCTCCTTGT-3′RP 5′-TAACAGTCTGAGATCCACCA-3′	171	59
**CM**	FP 5′-GTCTACACACCTGCCATTAG-3′RP 5′-GTACAAATCAGCAACTAGGC-3′	198	52
**PAL**	FP 5′-GCAAGATAGATACGCTCTAA-3′RP 5′-GTTCCTTGAGACGTCAAT-3′	136	49
**C4H**	FP 5′-GCAACATTGATGTTCTCAAC-3′RP 5′-TCCAGCTCTTCAAGGACTAT-3′	169	53
**GS**	FP 5′-TGGGTAGATTAGAAGCCAGA-3′RP 5′-CTGGTGATTTCTACCAGCTC-3′	139	52
**G10H**	FP 5′-TATCGAGCTTTTCAGTGGAT-3′RP 5′-GATGTGAGTCCTGTCGATTT-3′	136	52
