# Supplementary material for: Exogenous feeding of immediate precursors reveals synergistic effect on picroside-I biosynthesis in shoot cultures of Picrorhiza kurroa Royle ex Benth
Source: Sci Rep. 2016 Jul 15;6:29750. doi: 10.1038/srep29750 (PMC4945949; doi:10.1038/srep29750)
Supplement: Supplementary Information [file srep29750-s1.doc]

**Exogenous feeding of immediate precursors reveals synergistic effect on picroside-I biosynthesis in shoot cultures of *Picrorhiza kurroa* Royle ex Benth**

Varun Kumar, Neha Sharma, Hemant Sood & Rajinder Singh Chauhan*

Department of Biotechnology and Bioinformatics, Jaypee University of Information Technology, Waknaghat- 173234, Solan (HP), India

**Supplementary figure legends**

**Supplementary Fig 1.** HPLC profiles of P-I standard and samples.

**Supplementary Fig 2.** HPLC profiles of p-CA standard and samples.

**Supplementary Fig 3.** HPLC profiles of CA standard and samples.

**Supplementary Fig. 1.**


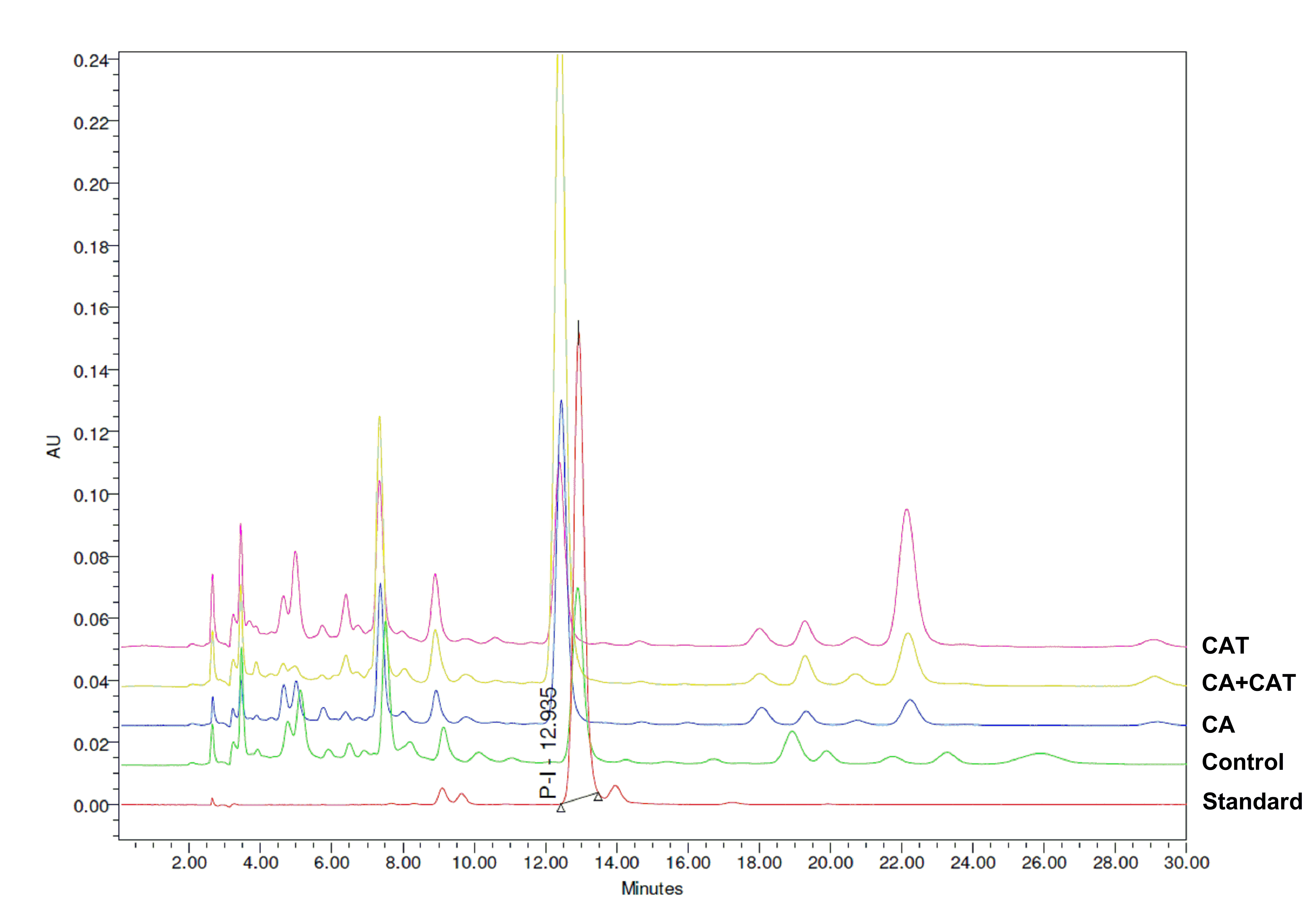


**Supplementary Fig. 2.**


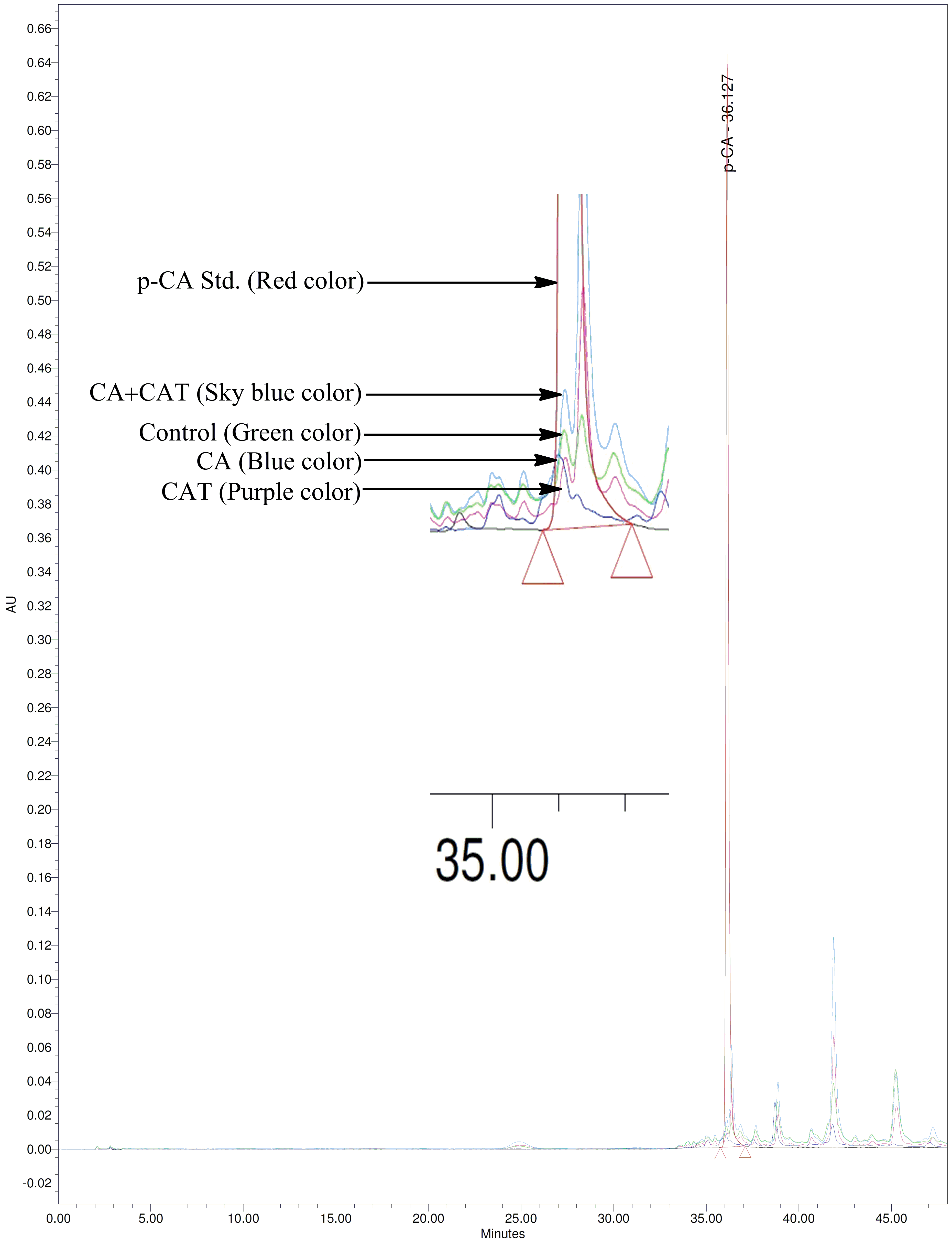


**Supplementary Fig. 3.**

**
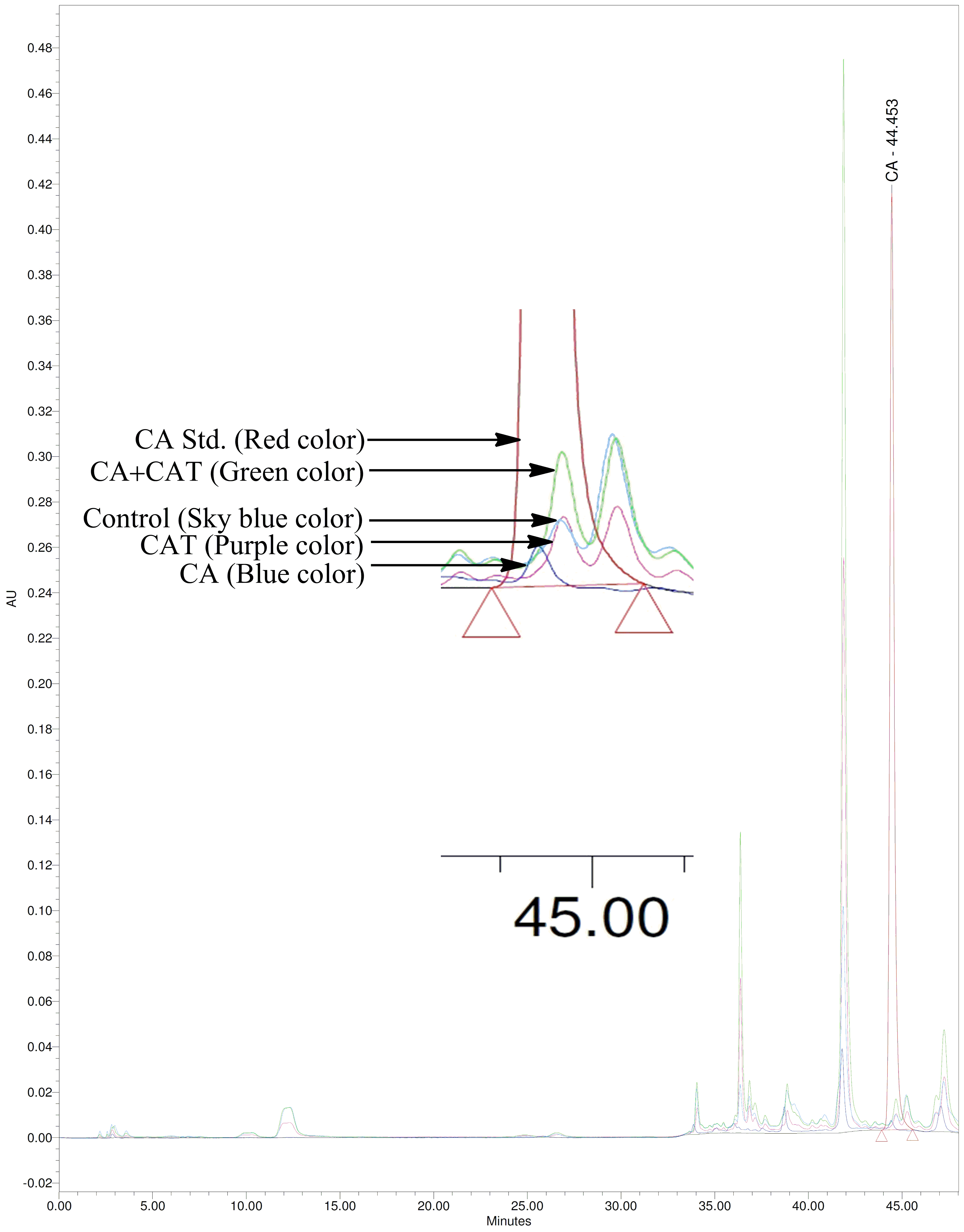
**
